# Intrauterine intestinal volvulus without malrotation presenting neonatal abdominal compartment syndrome

**DOI:** 10.1016/j.ijscr.2022.107742

**Published:** 2022-10-11

**Authors:** Hirokazu Matsushima, Morihiro Katsura, Masafumi Ie, Ryuichi Genkawa

**Affiliations:** a Department of Surgery, Okinawa Chubu Hospital, 281, Miyazato, Uruma, Okinawa 904-2293, Japan; b Department of Neonatology, Okinawa Chubu Hospital, 281, Miyazato, Uruma, Okinawa 904-2293, Japan

**Keywords:** Intestinal volvulus, Pediatric surgery, Obstetrics, Neonatology

## Abstract

**Introduction:**

Fetal intestinal volvulus without malrotation is extremely rare, and early prenatal diagnosis is challenging because the signs and symptoms are non-specific. However, without proper management, it can cause massive bowel necrosis.

**Presentation of case:**

A woman experienced a dilated fetal bowel at 34 weeks of pregnancy and noticed a decrease in fetal movements at 36 weeks; however, she did not visit a hospital. Her newborn developed severe abdominal distension and was diagnosed with neonatal abdominal compartment syndrome with respiratory distress immediately after emergency caesarean section at 36 weeks and 5 days of pregnancy. The neonate underwent emergency exploratory laparotomy. This revealed a volvulus of the small bowel with extensive necrosis and no findings of congenital malrotation. While the patient required massive necrotic bowel resection, 80 cm of the small intestine was preserved.

**Discussion:**

Fetal intestinal volvulus without malrotation can cause abdominal compartment syndrome with rapid respiratory distress. Therefore, it should be considered in the differential diagnosis of fetal intestinal dilatation. Volvulus exacerbation risk increases from 30 weeks of pregnancy to late preterm delivery. However, the time lag between the mother's awareness of decreased fetal movement and caesarean section makes early diagnosis challenging, resulting in a life-threatening condition for the neonate.

**Conclusion:**

When a fetal ultrasound examination shows intestinal dilatation between gestational week 30 and late preterm, the mother must be fully informed about the possibility that the foetus has intestinal volvulus and the potential risk of massive fetal intestinal necrosis.

## Highlights

• Volvulus without malrotation should be considered in fetal bowel dilatation.
• This can cause abdominal compartment syndrome, resulting in respiratory distress.
• Fetal bowel dilatation without polyhydramnios is a key finding for early diagnosis.
• There is a risk of massive fetal bowel necrosis around the late preterm period.
• Early recognition and informed consent are essential for a good neonatal prognosis.

## 1 Introduction

Intestinal volvulus without malrotation is one of the differential diagnoses of fetal intestinal dilatation [1]. This is an extremely rare condition known to cause abdominal compartment syndrome in newborns [2], [3], [4]. A clear aetiology is yet to be established [5], [6], and early prenatal diagnosis is difficult because the signs and symptoms are non-specific [5], [6]. When improperly managed, it can cause massive bowel necrosis and peritonitis [6]; therefore, close communication and cooperation between obstetricians, neonatologists, surgeons, and the mother is important before the affected baby is born. We herein present a case of intestinal volvulus without malrotation leading to abdominal compartment syndrome in a neonate who underwent emergent exploratory laparotomy. We also discuss the clues involved in their emergent diagnosis and perinatal management. This case report was written in line with the SCARE 2020 criteria [7].

## 2 Presentation of case

**Fig. 1:**
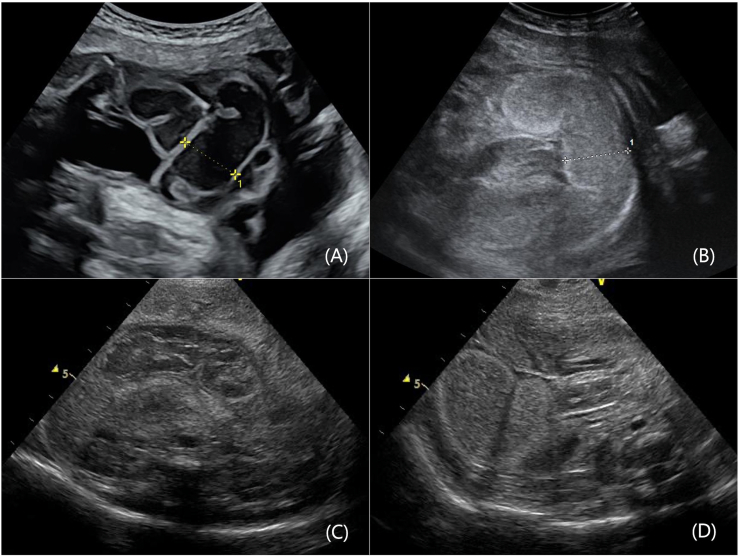
Prenatal ultrasound examination of the fetal abdomen shows intestinal dilatation of 24 mm and thickening of the intestinal wall during a prenatal checkup at 34 weeks of pregnancy (A), and intestinal dilation worsening of the diameter to 26 mm and the high intestinal brightness at 36 weeks and 5 days of pregnancy (B). Postnatal abdominal ultrasound examination shows dilated bowel loops and thickening of the intestinal wall (C, D), whereas meconium is detected from the sigmoid colon to the rectum without ascites (D).

A 30-year-old mother in her second pregnancy was referred to our institution for a fetal abnormality. During a prenatal checkup at 34 weeks of pregnancy, fetal ultrasound examination showed a mild, 24-mm intestinal dilatation without polyhydramnios (Fig. 1a). The obstetrician considered that an emergency caesarean section was not warranted at this time because the intestinal dilatation was mild, and there were no findings of fetal distress. At 36 weeks of pregnancy, the mother noticed decreased fetal movement but did not visit a hospital. A second fetal ultrasound examination revealed intestinal dilatation worsening with poor mobility during the prenatal checkup at 36 weeks and 5 days of pregnancy (Fig. 1b). The fetal heart rate labour diagram showed loss of fetal heart rate baseline variability and repeated late decelerations. The mother was diagnosed with fetal distress, and an emergency caesarean section was performed.

**Fig. 2:**
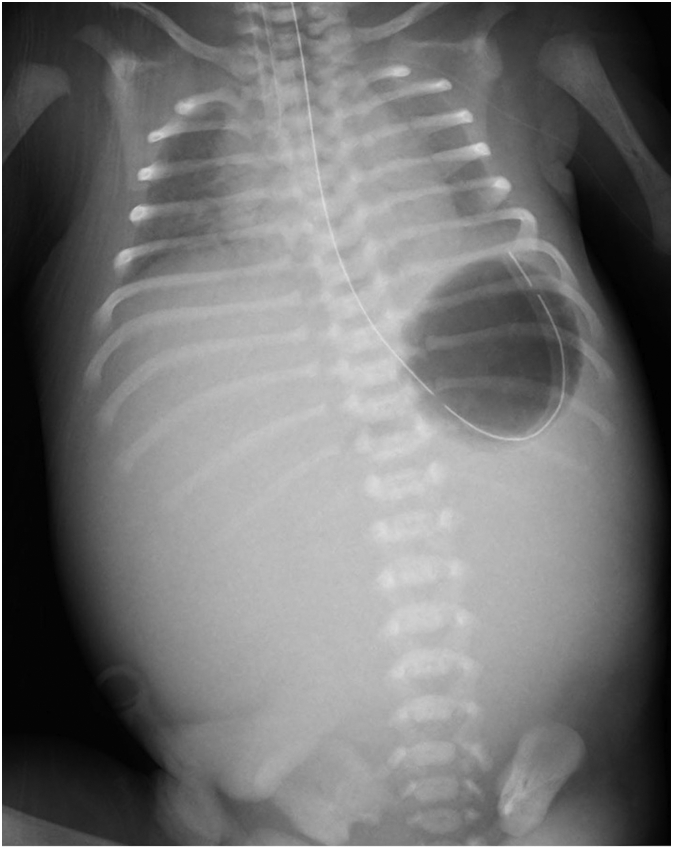
Postnatal abdomen supine X-ray shows stomach gas without bowel gas passage and prominent diaphragmatic elevation.

**Fig. 3:**
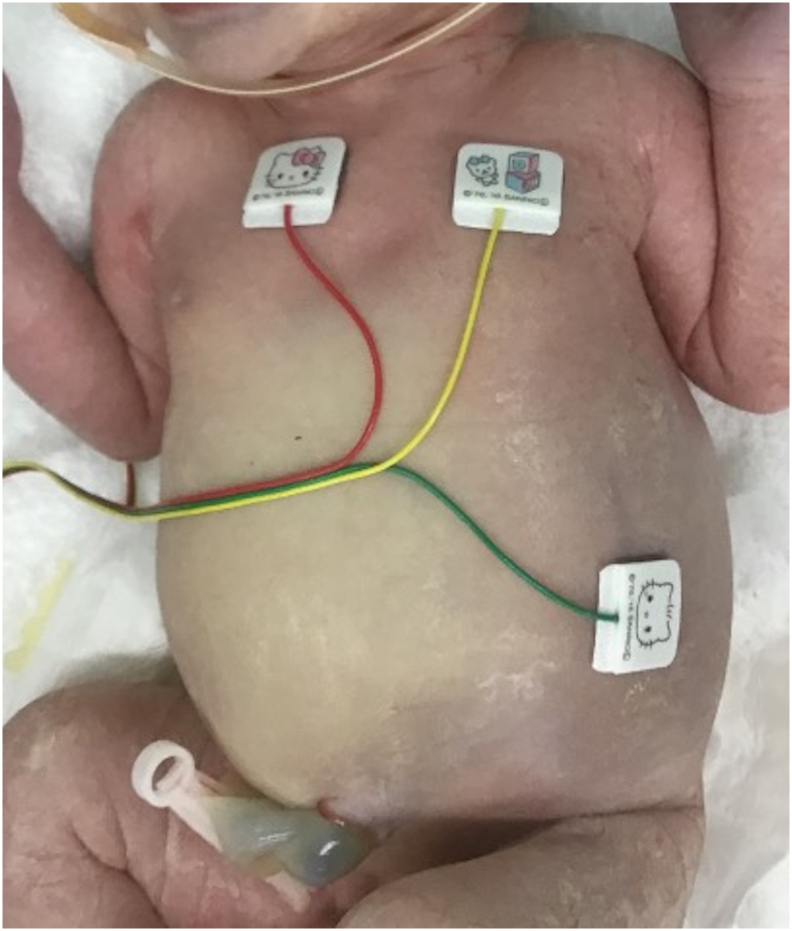
Physical examination reveals severe abdominal distention showing bluish-white skin discolouration of the abdominal wall.

A male infant weighing 2652 g was delivered with Apgar scores of 5 and 7 at 1 and 5 min after birth, respectively. A gastric tube was secured in the gastric fundus (Fig. 2), and bilious drainage was detected. Physical examination revealed severe abdominal distension with diminished bowel sounds, generalised oedema, and bluish/white skin discolouration of the abdominal wall (Fig. 3). The infant passed meconium through the anus immediately after birth. Vital signs at birth were not very unstable (body temperature of 37.1 °C, blood pressure of 42/18 mmHg, pulse of 170/min, and respiratory rate of 42/min). However, the patient's respiratory function was impaired despite using a continuous positive airway pressure mask because of the markedly increased intra-abdominal pressure. The newborn was intubated in the NICU and was placed on mechanical ventilation.

Initial laboratory values included a white blood cell count of 12.3 × 109 cells/μL, haemoglobin levels of 9.9 g/dL, and platelet count of 25.8 × 104 cells/μL. Blood gas analysis revealed the following: pH, 7.31; PO_2_, 27.9 mmHg; PCO_2_, 45.7 mmHg; HCO_3_^−^, 22.3 mmol/L; lactate, 3.0 mmol/L. A plain radiograph revealed the presence of gastric bubbles without intestinal gas in the abdomen (Fig. 2). Abdominal ultrasound examination results indicated segmental intestinal dilatation and thickening of the intestinal wall (Fig. 1c). Meconium was detected from the sigmoid colon to the rectum (Fig. 1d), and ascites was not present. Based on these examination results, the patient was diagnosed with abdominal compartment syndrome, resulting in rapid respiratory distress with bowel dilatation.

The differential diagnosis of abdominal compartment syndrome with bowel dilatation immediately after birth is meconium ileus, necrotising enterocolitis (NEC), and bowel perforation.

**Fig. 4:**
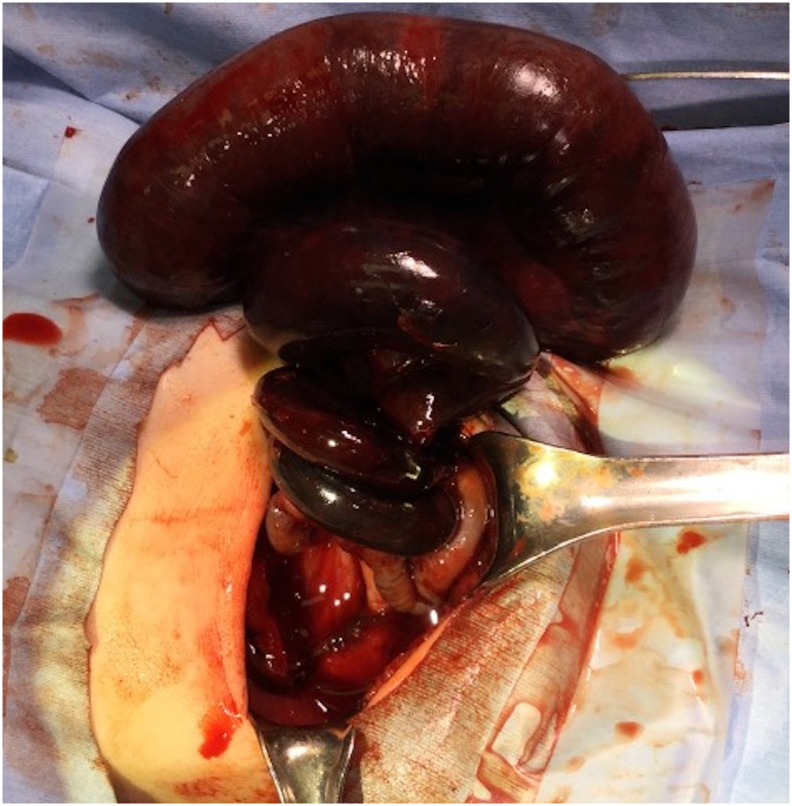
On laparotomy, the ileum was twisted about four-fold clockwise and showed extensive bowel necrosis.

The patient had abdominal compartment syndrome and underwent emergent exploratory laparotomy within a few hours after birth. Exploration revealed volvulus of the small bowel with extensive ischemia from 80 to 100 cm distal from the ligament of Treitz to proximal to the ileocecal valve, with haemorrhagic ascites (Fig. 3). The ileum was found to be twisted four-fold clockwise (Fig. 4). The positions of the caecum and ligament of Treitz were normal, and there were no findings of congenital malrotation. Therefore, the patient was diagnosed with intestinal volvulus without malrotation. Because the blood vessels in the ileocecal region showed thrombotic occlusion, we resected the extensive necrotic small intestine and caecum and performed end-to-end anastomosis between the jejunum and ascending colon. More than 80 cm of the remaining small intestine was preserved. The newborn was started on oral feeding on postoperative day 4 and extubated on postoperative day 8. The newborn was discharged on postoperative day 22 with full oral feeding after confirming weight gain.

## 3 Discussion

Fetal intestinal volvulus without malrotation is extremely rare, and no clear aetiology has been identified thus far [5], [6]. This case posed difficulties in early diagnosis and determination of the optimal delivery timing.

The signs and symptoms of this disease include decreased fetal movement, fetal heart rate labour diagram abnormalities, intestinal dilatation and the whirlpool sign on fetal abdominal ultrasound examination [6], [11], [12]. However, early prenatal diagnosis in the absence of fetal intestinal ischaemia is difficult because all these findings are non-specific and can manifest when intestinal necrosis has already started. Cases of congenital intestinal atresia may show polyhydramnios [6], [11], but this sign is rarely seen in intestinal volvulus without malrotation. It has been reported that amnion fluid is reabsorbed in the first 25–35 cm of the jejunum, and polyhydramnios is associated with high intestinal atresia [11]. Because the present case did not involve polyhydramnios, we expected the site of intestinal obstruction to be more distal than the ileum preoperatively if the neonate had congenital intestinal atresia or stenosis. In this case, a further reason for the lack of polyhydramnios was the rapid exacerbation of intestinal volvulus in the late preterm period [13]. Moreover, the presence of meconium after birth is an atypical finding in congenital intestinal atresia.

No well-defined diagnostic criteria exist for abdominal compartment syndrome in neonates [4]. Neonatal abdominal compartment syndrome has a high mortality rate (29 %), and early surgical treatment is usually required [4]. In this case, the patient's haemodynamic status was not unstable, but the newborn had severe abdominal distention leading to prominent respiratory distress; therefore, we made a clinical diagnosis of abdominal compartment syndrome. A PubMed search as of February 2022 revealed nine previous case reports of this disease; we summarised their characteristics (Table 1). Most previous case reports also demonstrated abnormalities in fetal vital signs, mainly concerning respiratory rather than circulatory features [5], [6], [12], [14], [15], [20]; the possibility of abdominal compartment syndrome needs to be considered when respiratory distress is observed in addition to abdominal distention in neonates. Meconium ileus, necrotising enterocolitis (NEC), and bowel perforation should be considered as the differential diagnoses that can cause abdominal compartment syndrome immediately after birth [2], [3], [4], [8], [9], [10].

**Table 1:** Summarize of previous care report of fetal intestinal volvulus without malrotation.

Author name, publication year	Weeks of caesarean section	Time interval between awareness of decreased fetal movement and caesarean section	Presence of polyhydramnios	Apgar score (1 min/5 min)	Respiratory distress
Nagano A, 2021 [4]	35 weeks and 2 days	1 day	Unknown	6/7	+
Hara K, 2015 [14]	35 weeks and 6 days	About 6 days	Unknown	9/10	+
Nakagawa T, 2015 [17]	34 weeks and 4 days	2 days	Unknown	6/8	Unknown
Jakhere SG, 2014 [18]	35 weeks and 4 days	Unknown	Unknown	8/10	−
Chung JH, 2013 [5]	37 weeks and 3 days	3 days	No	5/6	+
Park JS, 2008 [12]	33 weeks and 2 days^a^	Unknown	Unknown	8/9	+
Morikawa N, 1999 [15]	31 weeks and 6 days	2 days	No	1/4	+
Di Maggio G, 1997 [19]	27 weeks	2 days	No	9/9	−
Usmani SS, 1991 [20]^b^	29〜30 weeks	Unknown	Yes	Unknown	Unknown
	34 weeks	Unknown	No	1/3	+

a The newborn was delivered transvaginally.

b This previous care report describes two cases of fetal intestinal volvulus without malrotation.

Intestinal volvulus without malrotation is associated with a high risk of massive bowel necrosis and peritonitis in neonates [6]. However, only 26 % of foetuses with intestinal dilation have abnormalities after birth [16]. On prenatal ultrasound examination, fetal bowel dilatation appears as fluid-filled intestinal loops greater than 7 mm. Therefore, congenital atresia and stenosis of the intestine should be prioritised when ultrasound examination at the late preterm stage of pregnancy shows intestinal dilatation greater than 14.5 mm in diameter [1]. Furthermore, the fetal heart rate labour diagram is the only objective indicator of fetal condition. Based on previous reports, most patients were born by emergency caesarean section between gestational week 30 and late preterm, with a time lag between the mother's awareness of decreased fetal movement and caesarean section surgery (Table 1) [5], [14], [15], [17], [18], [19], [20]. In the case of intestinal dilatation greater than 14.5 mm in diameter during prenatal checkups, the fetal intestine may become ischaemic, and the fetal heart rate labour diagram must be frequently checked. Therefore, performing an immediate caesarean section and emergency fetal surgery when necessary is imperative. In this case, although the mother was aware of decreased fetal movement at 36 weeks of pregnancy, she did not come to the hospital on her initiative until the regular prenatal checkup. If the mother had been aware of the severity of the fetal conditions, she would have visited the hospital immediately after noticing decreased fetal movement; this could have led to earlier diagnosis and surgical intervention.

Finally, although fetal intestinal volvulus without malrotation is extremely rare [5], signs of this disease include intestinal dilatation without polyhydramnios on fetal ultrasound examination and the presence of abdominal compartment syndrome immediately after birth. An infant born with this disease could develop massive bowel necrosis and peritonitis with abdominal compartment syndrome [6]. Therefore, if intestinal dilatation is detected during a prenatal checkup, the affected fetal intestine should be carefully monitored, and the possibility of ischaemic necrosis should always be considered. Although no consensus exists regarding optimal delivery timing, based on our case and previous reports, the risk of volvulus exacerbation increases from 30 weeks of pregnancy to late preterm delivery (Table 1) [5], [12], [14], [15], [17], [18], [19], [20]. Therefore, collaboration among obstetricians, neonatologists, and pediatric surgeons, and decision-making for optimal delivery shared with the mother based on accurate informed consent are important to achieve good outcomes.

## 4 Conclusion

Fetal intestinal volvulus without malrotation is extremely rare. However, in the case of fetal intestinal dilatation without polyhydramnios, this condition should be considered in the differential diagnosis. It can cause abdominal compartment syndrome resulting in rapid respiratory distress. When a fetal ultrasound examination shows intestinal dilatation, the mother needs to be fully informed about the possibility that the foetus may have congenital intestinal atresia or volvulus, which usually requires emergency surgery for both the mother and the newborn. In addition, the doctors and the mother must be aware of the potential risk of massive fetal intestinal necrosis between gestational week 30 and late preterm when a fetal ultrasound examination shows intestinal dilatation.

## Funding

This study was supported by Okinawa Chubu Hospital Alumni Association grant.

## Ethical approval

It is not required.

## Consent

Written informed consent was obtained from the patient to publish this case report and accompanying images. On request, a copy of the written consent is available for review by the Editor-in-Chief of this journal.

## Author contribution

MK and HM contributed to the conception, the acquisition, interpretation of data, drafting the work and revising it critically for important intellectual content. MI and RG were actively involved in decision making and patient treatment and revising the draft critically for important intellectual content. All authors approved final version to be published.

## Research registration number

Not applicable.

## Guarantor

Morihiro Katsura.

## Provenance and peer review

Not commissioned, externally peer-reviewed.

## Declaration of competing interest

The authors have not declared a specific grant for this research from any funding agency in the public, commercial or not-for-profit sectors.
